# Land-use and land-cover assessment for the study of lifestyle change in a rural Mexican community: The Maycoba Project

**DOI:** 10.1186/1476-072X-11-27

**Published:** 2012-07-09

**Authors:** Mario A Giraldo, Lisa S Chaudhari, Leslie O Schulz

**Affiliations:** 1 Department of Geography and Anthropology, Kennesaw State University, 1000 Chastain Rd., Keenesaw, GA, 30144, USA; 2 College of Health and Human Services, Northern Arizona University, PO Box 15015, Flagstaff, AZ 86011, USA

**Keywords:** Land-use change, GIS, Remote sensing, Lifestyle, Diabetes, Mexican Pimas, Mexico

## Abstract

**Background:**

In 1995, a study was conducted to identify the effects of traditional and westernized environments on the prevalence of type 2 diabetes in Pima Indians (Pimas) in Mexico and the United States. The study concluded that the more traditional lifestyle in Mexico had a protective effect against this metabolic disorder. In the ensuing 15 years, the environmental circumstances of the Mexican Pimas changed, and a follow-up study was conducted to determine the role environmental change plays in the development of diabetes in this genetically susceptible population. A major element of environmental transition relates to land-use and land-cover (LULC) changes that could affect physical activity and promote an obesogenic environment. This study examined changes in the region’s LULC to determine whether there have been transitions in agricultural land use and urbanization that would be consistent with a more sedentary lifestyle. Changes were assessed from 1994 aerial photographs and 2007 satellite images.

**Results:**

The land-cover analysis showed that mixed vegetation and dense trees cover most of the study area. It suggested a rural environment that includes a low percentage of impermeable areas, and it indicated that the area experiencing human intervention covers 7% of the total area. The land-use-change findings showed a decrease or no change in agricultural or ranching areas and a decrease in farmland due to reforestation or revegetation. Three variables from the land-use-change analysis were examined as proxies for lifestyle change: urban development, dwelling-unit density, and variation in the road network. Two of the measures –the amount of urbanization and the number and density of dwelling units—showed increases, most notably in the town of Maycoba. There were only minor changes in the road network: most of the road segments are short and concentrated in Maycoba where most of the buildings, points of interest (e.g., church, stores), and cars are located.

**Conclusions:**

The LULC in Maycoba and surrounding settlements had changed during the study period**.** LULC change was used as a proxy to examine lifestyle changes that can affect levels of physical activity.

## Background

In 1995, a cross-sectional study was conducted to identify the effects of traditional and westernized environments on the prevalence of type 2 diabetes in Pima Indians (Pimas) in Mexico and the United States [1-3]. The two populations share the same linguistic family group and are genetic relatives; therefore, are presumed to have the same genetic risk for diabetes [4]. Their living environments, however, are dramatically different. The Mexican Pimas live in the remote village of Maycoba in the Sierra Madre Mountains, and in 1995, they had experienced relatively little change in their traditional lifestyle. At that time, nothing was known about the prevalence of diabetes in their society. In contrast to the Mexican Pimas, the U.S. Pimas live in a westernized society and are known to have one of the world’s highest rates of diabetes [5,6]. An important finding of the 1995 study was that the prevalence of diabetes was significantly lower in the Mexican Pimas than in U.S. Pimas despite their similar genetic predisposition. The study concluded, therefore, that the more traditional lifestyle in Mexico had a protective effect against this metabolic disorder [2].

Over the ensuing 15 years, the environmental circumstances of the Mexican Pimas changed, and a follow-up study was conducted to determine the role environmental change plays in the development of diabetes in this genetically susceptible population. A major element of environmental transition relates to the changes in land-use because those changes could affect physical activity and promote an obesogenic environment, which are influencing factors in the development of diabetes [7]. Agrarian community members are expected to have high level of interaction with their local environment. When modernization of the local economy occurs, a transformation of the local setting can be seen in its land-use. This transformation is expected to bring changes in lifestyle of the local residents [8]. Therefore, a component of the follow-up study was devoted to examining the region’s land-use and land-cover (LULC) and changes in land-use to determine whether there have been transitions in agricultural land use and urbanization that would be consistent with a more sedentary lifestyle.

Health and medical geography studies recognize the importance of place and the interaction between people and place with regards to health, disease, and health care [9]. The use of remote sensing in health studies offers an additional viewpoint for a multilevel analysis: gather systematic environmental data and perform environmental change analyses. This is a key element since a traditional lifestyle is linked to a close relationship with the environment. Although a number of important environmental changes have been observed in the study area, they have yet to be analyzed in a standardized manner afforded by remote sensing and geographic information systems (GIS). In this context, the combined analysis of LULC change, population growth, and urban and infrastructure development support the surveillance of diabetes and obesity by identifying changes in the local setting linked to lifestyle that contribute to clinical observations and findings. Using aerial photographs and satellite images that represented LULC in 1994 and 2007, the current study determines whether there have been transitions in agricultural land use and urbanization that would be consistent with a more sedentary lifestyle.

## Methods

### Study area

**Figure 1:**
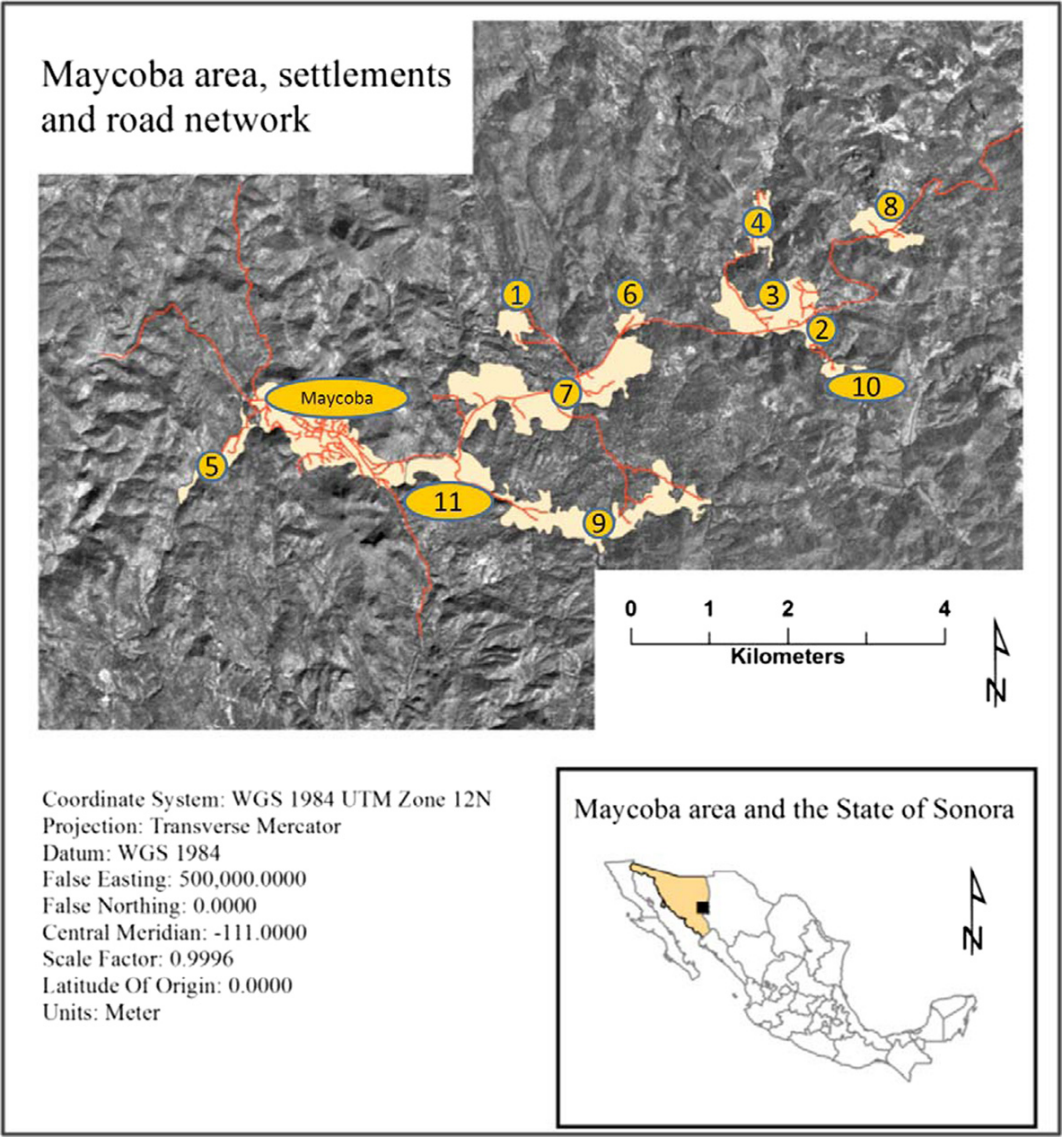
**Location of the study area: Maycoba and surrounding settlements within the state of Sonora, Mexico.** (Numeric labels in this figure correspond to the numbers of the settlement listed in Table 3 and Figure 3).

The study area includes the rural town of Maycoba and 11 surrounding communities, located in Sierra Madre Occidental in the state of Sonora, Mexico (Figure 1).

LULC and land-use changes were studied using aerial photographs from 1994 and satellite images from 2007. All image analysis was performed using ERDAS 2010 (Leica Geosystems), and the GIS analysis was done using ArcGIS 9.3 (ESRI).

### Aerial photographs

The aerial photographs were obtained from Mexico’s National Institute of Statistics and Geographic Information (INEGI) in Hermosillo, Mexico. These images are from October 1994. They are in tag image file format (TIFF) files with a 1m pixel size, a 1:20000 scale, and they were taken as consecutive images in the flight line. The 2007 satellite images were used to geometrically correct and geo-reference the 1994 aerial photographs. The 1994 land-use maps were created using ArcGIS. Vector files were created for four variables: human settlements, dwelling units, road network, and agricultural fields. Land-use parcels were digitized around homogeneous areas with a single land use and limited by visible boundaries such as a stream, trail, or road [10].

### Satellite images

The 2007 satellite images were obtained from Geoeye, a U.S. company headquartered in Colorado. These images are in the form of Ikonos-2 multispectral and panchromatic files. Two additional subset satellite images in the same format were acquired to capture the entire study area. The multispectral files have a 3.6 m spatial resolution; the panchromatic files have a 0.9 m spatial resolution. Mosaics were initially created using the Ikonos-2 panchromatic files to visually inspect the spectral variation of the different land-use classes between the two image sets [10,11].

These 2007 satellite images were analyzed for land-cover by creating a land-cover map of the area. This was generated using the multispectral data from the satellite images in ERDAS 2010 (Leica Geosystems). The land-cover was classified into five categories (see below). The classification of satellite data followed a supervised approach consisting in the collection of sampling signatures for each one of the land-uses. To account for the land-cover heterogeneity 20-30 signatures were collected for each land-cover class. The signature file was used to conduct the image classification. Using the recode function the final classified images were recoded to the final five classes. The initial accuracy of the classification was verified by selecting 30 random points per class. Additional signatures were collected for classes with low accuracy and an updated signature file was used to repeat the classification. The classification process was repeated until the recoded image had an accuracy level of more than 90% for all the classes. The Kappa coefficient of agreement and a confusion matrix were prepared to determine accuracy (see Table 1 and below). The land cover classification analysis presents an overall accuracy of 94.2%. An additional accuracy assessment of this process was also verified during fieldwork in 2011 (see section below).

**Table 1:** Confusion matrix for the land-cover assessment

	**Reference data**					
	**Trees**	**Mixed vegetation**	**Non- vegetated surfaces**	**Water**	**Shadow**	**Row total**
**Trees**	28	2	0	0	0	30
**Mixed vegetation**	4	26	0	0	0	30
**Non-vegetated surfaces**	0	0	30	0	0	30
**Water**	0	0	0	30	0	30
**Shadow**	0	0	0	1	29	30
**Column total**	32	28	30	31	29	150
**Overall accuracy**	(28 + 26 + 30 + 30 + 29)/150 = 95.3%					

**Table 2:** Change in parcels under human intervention between 1994 and 2007

**Land-use categories**	**1994**		**2007**		**Percent change in area (ha)**
	**Number of parcels**	**Sum Area (ha)**	**Number of parcels**	**Sum Area (ha)**	
Agriculture	42	302.0	38	258.0	−14.6
Forest	23	65.6	35	122.0	85.9
Mixed vegetation	26	141.8	29	121.4	−14.4
Urban	1	24.0	1	36.4	51.6
Totals	92	533.4	103	537.8	0.8

**Table 3:** Change in areas under human intervention between 1994 and 2007

**Settlement identifier**	**Percentage change**	
	**Area(ha)**	**Perimeter(km)**
Maycoba	18.8	8.1
1	−35.5	−29.0
2	−10.1	−8.0
3	−5.2	−6.4
4	−6.1	0.0
5	0.0	0.0
6	12.7	−6.3
7	−3.0	−2.0
8	6.7	−3.6
9	−5.0	4.4
10	0.0	0.0
11	−2.4	−11.4
Total	0.5	−1.6

The Kappa coefficient was calculated using the following equation:

(1) $$K=\frac{N\sum_{i=1}^{k}X_{\mathrm{ij}}-\sum_{i=1}^{k}\left(x_{i}*x_{j}\right)}{N^{2}-\sum_{i=1}^{k}\left(x_{i}*x_{j}\right)}$$

(2) $$\begin{array}{l} N=150\\ \sum_{i-1}^{k}X_{\mathrm{ij}}=\left(28+26+30+30+29\right)=143 \end{array}$$

(3) $$\begin{array}{l} \sum_{i=1}^{k}\left(x_{i}*x_{j}\right)=\left(30*32\right)+\left(30*28\right)+\left(30*30\right)+\left(30*31\right)+\left(30*29\right)=4500\\ K=\left(16950/18000\right)=94.2\% \end{array}$$

**Figure 2:**
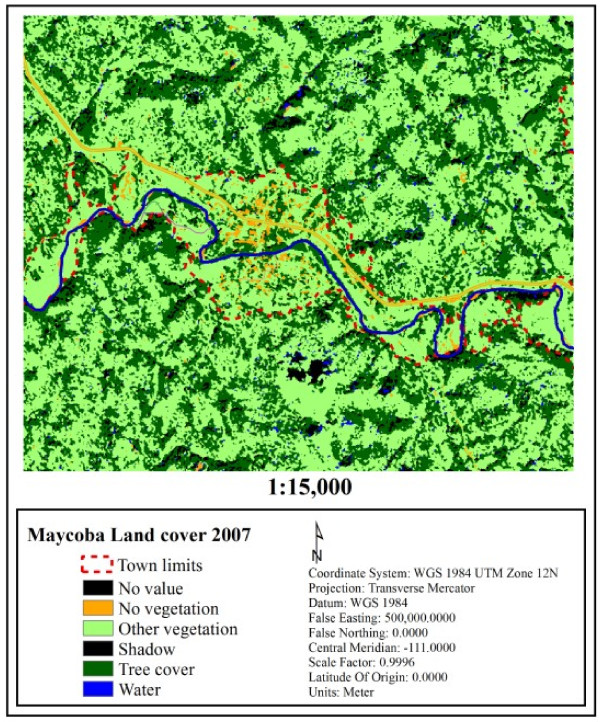
Land-cover classification of the study area.

For the landscape land-cover assessment the images were classified and recoded as five classes (see Figure 2):

(1) tree cover: areas under tree cover with a closed canopy

(2) mixed vegetation: areas with scattered trees and covered with grass, shrubs or agricultural fields

(3) non-vegetated surfaces: areas that produced no vegetation signature, including impermeable surfaces, human structures, roads, and sand banks

(4) water: rivers, ponds, and flooded areas

(5) shadow: areas covered by shadows of clouds, hills, or dense tree patches

The 2007 land-use maps were created using ArcGIS. Vector files were created and land parcels were digitized following the same process applied to the 1994 photographs. For the land-use analysis, the high spatial resolution of the data allows for high accuracy in the delineation of vector files. Since the remote sensing data was from 2007, the vector files were reviewed during the 2011 fieldwork to guarantee their accuracy. The land-use change analysis was performed using remote sensing analysis at the community level. The land-use-change analysis between 1994 and 2007 was centered on four variables: human settlements, dwelling units, road network, and agricultural fields.

A set of limitations deriving from this method requires discussion since it poses potential problems in analysis. Errors in the interpretation of high resolution remote sensing data are mostly due to human factors that include the lack of training in differentiating manmade structures, natural features, and in seeing variation between vegetation groups. The analyst may also produce errors during the on-screen delineation of polygons and lines by unintentionally modifying the original shape of ground features or by adding inexistent features to the new data layers. In this study, human error was minimized by having an experienced and trained analyst conducting the image interpretation; by selecting classes that have strong differences in their spectral signature and visual characteristics (i.e. forest, agriculture fields, houses, and roads), and by field verification including assistance from field guides and other investigators familiar with the study site.

In the land-use delineation, the layers created for the 1994 dataset were used as base maps for the delineation of the 2007 dataset. Co-registration of both datasets minimized spatial error. The change in shape and area accounts for the land-use change at the polygon level. In the quantification of household structures, this research does not verify how many families inhabit a house, the number of residents per house, or temporary residency.

### Fieldwork

In August 2011, fieldwork was completed to validate the LULC-analysis findings. The goal of the fieldwork was to determine the accuracy of the remotely conducted LULC analysis by verifying the results of a set of predetermined points from the LULC study. The fieldwork included walking throughout the study area, and taking field notes and photographs with the key assistance of local guides. The objective was to either corroborate the LULC findings or determine if the assessment needed to be revised. The fieldwork supported the LULC findings.

## Results

### Land-cover analysis

Results of the land-cover analysis showed that 57% of the study area (7402 hectares) is mostly under mixed vegetation that includes open canopy forest, bushes, grass, and areas exposed to human activity. Dense tree patches with closed canopies make up 36% of the total area. Impermeable areas, such as paved roads, open roads, sand banks, and dwelling units, account for less than 1.0% of the area (Figure 2).

A low percentage of impermeable areas suggests a rural environment where human settlements occupy relatively small areas when compared with the surrounding natural vegetation. Agricultural fields and cattle grasslands are concentrated in areas nearby or interwoven with human settlements, and they are easily differentiated from the surrounding landscape. Areas under human influence looked concentrated and appeared like islands within the surrounding vegetated landscape (Figure 2). Four percent of the total area cannot be classified due to terrain elevation and cloud cover that caused shadows in the images.

### Land-use analysis

For this analysis, land use is summarized into four categories (see Table 2):

(1) agriculture: ranching and cultivation activities

(2) forest: vegetation similar to the surrounding natural landscape

(3) mixed vegetation: presence of trees within cleared land

(4) urban: presence of buildings organized in blocks and interconnected by a series of streets.

According to the land-use analysis, the study area was divided into 12 different subareas where there has been human intervention. These are clearly delimited by the surrounding natural vegetation, indicating heterogeneity in the landscape (Table 2). The areas experiencing human intervention make up 7% (535 hectares) of the total area under study (7402 hectares). These areas were found on both sides of a seven-kilometer section of a paved interstate highway that bisects the study area.

### Land-use-change analysis

The land-use-change analysis indicates a number of important changes took place between 1994 and 2007 in Maycoba and the surrounding human settlements. Despite the individual variation between the subareas, the total area affected by human activity increased by only 0.5% (2.6 hectares) (See Table 2 and 3. The perimeter values represent changes in areas affected by human activity.) A small variation in the total area experiencing human intervention and the minimal variation in the perimeter of the subareas confirm that changes in land-use are taking place in areas that have been affected by human activity in the recent past. The perimeter has decreased in seven settlements, which suggests a process of revegetation of open land and a regression towards a natural landscape in small sections of the subareas.

Between 1994 and 2007 there was a decrease in total area in seven subareas, an increase in total area in three subareas, and no change in only two subareas. (Table 3). Noteworthy is the increase in the area of Maycoba, the largest human settlement in the area. The growth of Maycoba during the 13-year period represents 19% of the entire study-area growth. Based on these results, it is clear that change within the 12 settlements is heterogeneous.

As Table 2 indicates, there were increases in forest and mixed vegetation land-use categories and decreases in the number of agricultural parcels. The increase in the total number of parcels from 1994 to 2007 was relatively small (11 parcels). The urban class was assigned to only one parcel in the area of Maycoba because it had a higher density of houses and streets. A change in the total number of parcels indicates increasing fragmentation of the landscape and a dynamic change in land use. In fact, during the 13-year period covered by the analysis, the number of parcels under agriculture and mixed-vegetation classification decreased approximately 14% each, while the number of parcels under forest and urban classification increased by 85% and 52%, respectively. These results suggest changes in the economic activities in the region, specifically, decreases in agricultural activities such as cultivation and ranching and an active process of revegetation. These findings are in agreement with the findings in the previous section that reported revegetation occurring in parts of the study area.

**Figure 3:**
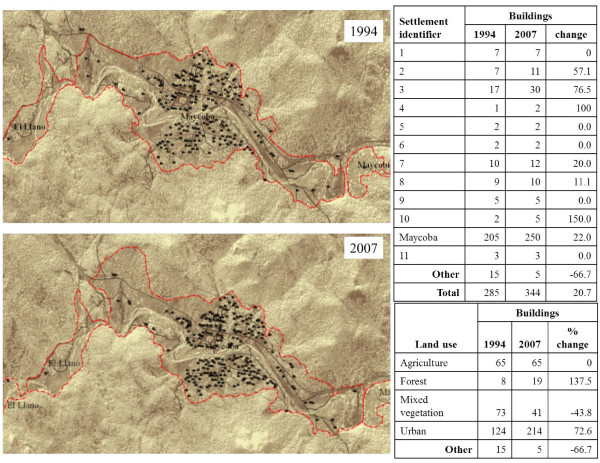
Change in urban development of the study area.

The land-use-change analysis explored three variables as proxies for lifestyle change: urban development, dwelling-unit density, and variation in the road network. Within the study area, the number of dwelling units (indicating urban development) had increased in seven settlements and had remained the same for five settlements (Figure 3). The highest density of dwelling units was found within the largest and most urban settlement of Maycoba. Forty-five new structures were observed, which represents a 22% increase in the Maycoba settlement or a 76% increase in structures for the entire study area between 1994 and 2007. These results indicate that the process of urban growth and increase in dwelling-unit density is mostly concentrated in the town of Maycoba.

In 2007 the road system of the study area included 69 kilometers of roads organized in 130 road segments. The maximum road length of a segment was 17 kilometers (the interstate highway), and the minimum road length of a segment was less than 100 meters. Image analysis determined that there were no major differences in the basic structure of the road system in 2007 when compared with the 1994 road network—with the exception of only few additions or better-defined streets within the urban areas. Streets and secondary roads (trails and pathways) intersect with the highway, which is the centralized main road of the study area. Maycoba is a denser settlement, where 94% of the streets are shorter than 400 meters (approximately four street blocks) and most of the buildings are found within a 400-meter radius of the town square. According to the fieldwork, there is a store, a bar, and a restaurant within a 200-meter radius of each major neighborhood in Maycoba.

The field study sample included 25 houses in Maycoba and the closest settlement east of this town. (This represented approximately 10% of the total). More than 15 of these dwelling units had a dedicated parking area for at least one car. Trucks were observed to be the most common vehicle in those households. Satellite dishes were also counted, and more than 13 houses had at least one satellite dish on their roof.

## Discussion

In relatively isolated rural communities where people still interact regularly with the natural landscape, LULC changes can suggest lifestyle changes linked to levels of physical activity [12]. The land-cover analysis shows that the study area is a relatively isolated rural community within a mostly naturally vegetated landscape. The absences of nearby larger towns or cities within the 7402 hectares of this study, the low percentage of total area under human settlements, the island-like quality of the small human settlements, and the low percentage of impermeable surfaces support this observation.

The most significant land-use changes found in the community of Maycoba are related to the decrease in the number of agricultural parcels, the increase in forested areas, and the growth of urbanized areas. These land-use changes suggest a transformation in the community’s living conditions and lifestyle from the one that existed in the 1990s. In the early 1990s, an ethnographic study of two Pima communities in the Sierra Madre Occidental 40 kilometers away, which are similar to the current study area and community considered here, showed that the community’s isolation made it relatively self-contained. Economic activities focused on subsistence agriculture, and surpluses were eventually traded with other communities [13]. The study reported that a forest was cleared and used as building material for housing and fences and that cleared fields were burned, plowed, and planted in a slash-and-burn agricultural system. Fallow fields allowed the fields to recover their natural fertility, but this system required adding new parcels to keep the system productive. They also reported that roads were unpaved and four-wheel vehicles were uncommon, which made walking and horseback riding the main forms of transportation within the community and among communities. Nonagricultural-related activities were rare due to the isolation of the communities and their focus on subsistence agriculture [13].

There was a transition in the local economy between 1994 and 2007, suggested by decreasing or untransformed cultivation and ranching areas and the associated active process of revegetation. These changes may have important implications that affect physical activity at the farm level. The natural process of revegetation of agricultural lands (decrease in agricultural parcels) suggests diminishing requirements for labor and other human activities. Even though there have been reforestation programs in the area [14] that are physically demanding during the early months of the growth cycle, the labor requirements are low in the following years. This is particularly true in this dry landscape where small amounts of rainfall limit tree growth rates [15].

In the last 13 years, the population growth from 1,191 to 1,316 inhabitants (based on censuses conducted during the 1995 and 2010 studies) and urban development of communities in the area of Maycoba have not been matched with the expected growth of cultivation and ranching areas for a subsistence-agriculture community. On the contrary, a heterogeneous reduction of these areas and revegetation were observed in the study area. Lack of growth in areas dedicated to cultivation and ranching suggest that these communities depend less on local agricultural production than they did before and thus community members spend less physical activity on subsistence activities.

On the other hand, businesses in the service sector, such as restaurants, bars, hotels, and stores, are more prominent in the residential areas than they were previously. In the 1990s, the lack of road infrastructure, the town’s isolation, and the subsistence-based economy offered opportunities for an active lifestyle. Currently, the increase in cars to access fields and other locations, as well as the plethora of satellite dishes, may have translated into a more sedentary lifestyle for residents.

Although the total area under human settlement has remained relatively unchanged, there has been an increase in the number of dwelling units (see Table 2 and Figure 3. Higher housing density increases the proximity between houses and points of interest like the church, school, and stores. Therefore, walking routines may be affected, and potentially, physical activity may be decreased [16,17]. Higher housing density also affects the relative size of the individual properties and the physical activities people perform in them. Larger properties allow activities such a gardening, cultivation, or livestock production. These activities are less demanding or feasible within smaller properties. Urbanization is associated with economic activities in the service sector and a transformation of the residents’ lifestyle.

Similarly, there has been no increase in improved road infrastructure and paved roads in areas under cultivation and ranching, and no decrease in the areas under forest cover that would suggest a focus on agricultural production. Rather, there has been an increase in the number and density of dwelling units within the observed communities. Paving the highway and some secondary roads after 1995 has brought important transformations to the local community. Paved roads make it easier to use motorized vehicles, which can affect walking patterns, particularly between farms and residential centers. This change has also allowed the development of new nonagricultural- related activities because it is easier to reach Hermosillo, the capital city of Sonora, and the neighboring towns of Yécora in Sonora and Yepachi in the state of Chihuahua.

This study found LULC changes have occurred in the study area from 1994 to 2007. Changes in the spatial setting that are reported in diabetes and obesity studies are usually associated to changes in lifestyle [18,19]. However, we are not suggesting that LULC changes are directly responsible for increasing diabetes in specific populations or asserting a cause effect relationship between LULC change and diabetes in this particular study. Assuming a cause and effect between these variables is an error described as ecological fallacy [20].

The critical element of place in health research and the use of geographic methods such as GIS in addressing these questions is increasingly integrated in health research [21,22]. Using remote sensing technology to assess the transformation of the physical space as a proxy to assess lifestyle change is in agreement with medical and health geography tradition examining the interplay between people and place in their experience with health and disease [23]. Agrarian communities are expected to have high level of interaction with the local environment. Therefore, the combined analysis of LULC change, urban and infrastructure development, and population growth, can be used to support the surveillance of diabetes at local and regional levels [16]. By identifying lifestyle change at the community level, GIS and remote sensing technologies complement, but does not substitute standard medical analyses conducted within the study population.

## Conclusion

In conclusion, land-use in Maycoba and the surrounding settlements has changed during the study period. These environmental changes, which include a decrease in the number of agricultural parcels and an increase in forested areas and urban growth, may influence levels of physical activity. A more sedentary lifestyle would be consistent with an increase in the prevalence of type 2 diabetes and obesity among the Pima Indians of Mexico. More information about the Maycoba Project can be found at the following website: http://www4.nau.edu/projectmaycoba[24].

## Competing interests

The authors declare they have no competing interests.

## Authors’ contributions

MAG led the study design and fieldwork conducted the data analyses, and assisted in writing the manuscript. LSC assisted in data acquisition, the fieldwork, data analyses, and in writing the manuscript. All authors reviewed, edited and approved the manuscript. LOS is the principal investigator of the study and led the manuscript preparation.
